# Patterns of violence and coercion with mental health among female and male trafficking survivors: a latent class analysis with mixture models

**DOI:** 10.1017/S2045796019000295

**Published:** 2019-05-30

**Authors:** L. Iglesias-Rios, S. D. Harlow, S. A. Burgard, B. West, L. Kiss, C. Zimmerman

**Affiliations:** 1 Department of Epidemiology, School of Public Health, Center for Midlife Science, University of Michigan, 1415 Washington Heights, Ann Arbor, Michigan 48109-2029, USA; 2 Department of Sociology, College of Literature Science, and the Arts, University of Michigan, 500 S State St, Ann Arbor, Michigan 48109, USA; 3 Survey Methodology Program, Institute for Social Research, University of Michigan, 46 Thompson St, Ann Arbor, Michigan 48104, USA; 4 Department of Global Health and Development, Gender Violence and Health Centre, London School of Hygiene and Tropical Medicine, 15-17 Tavistock Place, WC1H 9SH London, UK

**Keywords:** Gender, human trafficking, latent class analysis, mental health

## Abstract

**Aims:**

Human trafficking is a crime and a human rights violation that involves various and simultaneous traumatic events (sexual and physical violence, coercion). Yet, it is unknown how the patterning of violence and coercion affects the mental health of female and male trafficking survivors.

**Methods:**

We conducted a cross-sectional study using a sample of 1015 female and male survivors of trafficking who received post-trafficking assistance services in Cambodia, Thailand or Vietnam. We assessed symptoms of anxiety and depression with the Hopkins Symptoms Checklist and symptoms of post-traumatic stress disorder (PTSD) with the Harvard Trauma Questionnaire. Violence was measured with questions from the World Health Organization International Study on Women's Health. Latent class analysis (LCA) was used to identify distinct patterns of violence and coercion in females and males. Novel multi-step mixture modelling techniques were employed to assess the association of the emergent classes with anxiety, depression and PTSD in females and males.

**Results:**

LCA identified two distinct classes of violence and coercion experiences in females (class I: severe sexual and physical violence and coercion (20%); class II: sexual violence and coercion (80%)) and males (class I: severe physical violence and coercion (41%); class II: personal coercion (59%)). Females in class I had a two-fold increase in the odds of anxiety (OR = 2.10; 95% CI: 1.57–2.81) and PTSD (OR = 2.07; 95% CI: 1.03–4.17) compared with females in class II, but differences in the prevalence of anxiety, depression and PTSD were not significant when comparing males in class I to class II.

**Conclusions:**

Specific patterns of violence and coercion provide a more in-depth understanding of the role of gender in the experience of violence and coercion and its association with mental health in survivors of trafficking. This information could be useful to target comprehensive mental health services for female and male trafficking survivors.

## Introduction

Globally, estimates suggest that 28.7 million (71%) trafficked persons are women and girls and 11.6 million (28.9%) are men and boys (International Labour Office, 2017). Human trafficking is a crime and a human rights violation that frequently involves numerous forms of physical or sexual violence and psychological coercion, which can have long-term health consequences (Kiss *et al*., 2015; Ottisova *et al*., 2016).

Human trafficking tends to be gender-specific (e.g., females including children tend to be exploited more often for sex work or domestic work, men for fishing or construction) (International Labour Office, 2017; Zimmerman and Kiss, 2017) and survivors report experiencing multiple and heterogeneous acts of violence and coercion (Zimmerman *et al*., 2006; Kiss *et al*., 2015). However, the extent of research on survivors of trafficking tends to operationalise violence and coercion in terms of severity or as a binary event (Ottisova *et al*., 2016). A greater understanding of the patterning of a range of violence and coercion acts could provide an empirical basis to understand its impact on the mental health of female and male survivors and inform well-targeted service provision.

A previous systematic review of interpersonal patterns of violence in non-trafficked populations reported differences by sex (O'Donnell *et al*., 2017). Females were more likely to be defined by three classes that included sexual violence, emotional abuse and high exposure to multiple trauma types. Males were more likely to be in classes characterised by physical violence (O'Donnell *et al*., 2017). While there is currently no research on survivors of trafficking that has assessed the patterning of violence and coercion using a latent class analysis (LCA) approach, research indicates a high prevalence of violence in females ranging from 60% in the Study on Trafficking, Exploitation and Abuse in the Mekong sub-region (STEAM) (Kiss *et al*., 2015) to 90% in a multi-country European survey (Zimmerman *et al*., 2008). In males, violence was reported by almost one-third of survivors in a UK case records study (Turner-Moss *et al*., 2014) and by almost half of men (49.3%) in STEAM (Kiss *et al*., 2015). The prevalence of coercion through personal and family threats among trafficked people is reported to be approximately 50% to 80%, irrespective of sex (Zimmerman *et al*., 2006; Kiss *et al*., 2015). Our previous findings with the STEAM indicated that violence and coercion experiences during trafficking differ by sex and have important implications for mental health (Iglesias-Rios *et al*., 2018).

In this study, we first model violence and coercion as a multi-dimensional experience and allow for the possibility of gender-specific patterning. Second, we examine the association of the emergent profiles of violence and coercion (i.e., latent classes) with each mental health outcome (anxiety, Post-traumatic stress disorder (PTSD) and depression) for females and males.

## Methods

### Data source, study design and study sample

We conducted a cross-sectional analysis using data from the STEAM (Kiss *et al*., 2015). The study included 1015 trafficked males, females, youth and children (age 10–17 years) who attended post-trafficking assistance services in Cambodia, Thailand and Vietnam. The details of the study methodology have been published elsewhere (Kiss *et al*., 2015).

### Sample design

A two-stage sampling strategy was used to identify individuals using post-trafficking services. First, selected post-trafficking support services in Cambodia, Thailand and Vietnam were determined based on diversity of clientele (e.g., age, sex, sector of exploitation), service relationship with the International Office of Migration (IOM) country teams and agreements with government agencies (e.g., support, referral and service arrangements). Individuals in the sample were identified as trafficked by the local governmental and nongovernmental referral networks and post-trafficking service providers. Second, a consecutive sampling approach was used to invite all eligible individuals to participate in a structured interview after providing informed consent within 2 weeks of admission to the post-trafficking services between 2011 and 2013. Participants were recruited only if the on-site trained case or social worker determined that their participation would not cause harm to their well-being. The response rate for the baseline survey was 98%.

### Data collection and survey questionnaire

Interviewers (caseworkers or social workers) from the agencies providing post-trafficking services received an intense 1-week training provided by one of the principal investigators of STEAM (LK) in collaboration with the International Organization for Migration partners in each country. Ethical provisions were emphasised to ensure that participation was voluntary and confidential. The survey questionnaire was developed based on an instrument used in a previous European study on health and sex trafficking (Zimmerman *et al*., 2006). The questionnaire was adapted for the different study populations and the regions by the study team with input from the study interviewers. The survey included questions about sociodemographic characteristics, pre- and post-trafficking exposures and mental health outcomes. The instrument was translated into Khmer, Thai, Vietnamese and Lao in multiple steps: professional translation from English to other languages, group translation-discussion process with IOM counter-trafficking teams, pilot-testing and review after back-translation into English.

### Ethics

Ethical approval for the STEAM was granted by the London School of Hygiene and Tropical Medicine and by the corresponding ethical entities in Cambodia, Vietnam and Thailand. The present study was approved by the University of Michigan Health Sciences and Behavioral Sciences Institutional Review Board, eResearch ID: HUM00097096.

### Measures: anxiety, depression and PTSD measures

Anxiety and depression symptoms in the past week were measured by the symptom inventory Hopkins Symptom Checklist-25 (HSCL-25), a validated and widely used screening instrument (Derogatis *et al*., 1974; Mollica *et al*., 1987; Kleijn *et al*., 2001). It consists of 25 items: ten items for anxiety symptoms and 15 items for depression symptoms. The scale for each question includes four categories of response (‘Not at all’, ‘A little’, ‘Quite a bit’ and ‘Extremely’, rated 1–4, respectively). Two scores are calculated: the anxiety score is the average of all ten anxiety items, while the depression score is the average of the 15 depression items. The depression score is correlated with major depression as defined by the Diagnostic and Statistical Manual of the American Psychiatric Association, IV Version (DSM-IV) in several populations (Mollica *et al*., 1987; Kleijn *et al*., 2001). A cutoff of 1.625 instead of the established value of 1.7 was used to identify symptoms of depression, as item 12 in the questionnaire (i.e., loss of sexual interest or pleasure) was excluded, given the characteristics of the study population. For anxiety, we defined ‘symptomatic anxiety’ according to the conventional international cutoff score of 1.75 (Mollica *et al*., 1987).

PTSD symptoms in the past week were measured using the validated Harvard Trauma Questionnaire (HTQ) part IV, which includes 27 trauma symptoms (Mollica *et al*., 1992, 1996). Each question has four response categories: ‘Not at all’, ‘A little’, ‘Quite a bit’ and ‘Extremely’, rated 1– 4, respectively. A total score was calculated by averaging the 27 items. A cutoff of 2.0 was used to assess symptoms of PTSD based on previous research on trafficked individuals accessing post-trafficking services (Zimmerman *et al*., 2008; Hossain *et al*., 2010). The HTQ has been used extensively in cross-cultural settings and among Southeast Asian populations exposed to trauma (Mollica *et al*., 1992, 1993, 1996; Kleijn *et al*., 2001).

### Violence and coercion measures and covariates

To assess physical and sexual violence, validated questions from the World Health Organization (WHO) International Study on Women's Health and Domestic Violence were used (Garcia-Moreno *et al*., 2006). While these questions were not validated in the study population, they involved violent acts that are commonly reported by trafficked individuals in post-trafficking services and shelters (Hopper and Hidalgo, 2006; Hossain *et al*., 2010; World Health Organization and Pan American Health Organization, 2012). Participants were asked if while being trafficked anyone committed any of the following acts against them (yes/no):

1. Slapped you or threw something at you that could hurt you
2. Pushed or shoved you
3. Hit you with a fist or with something else that could hurt you
4. Kicked, dragged or beat you up
5. Tied or chained you
6. Choked you on purpose
7. Burned you on purpose
8. Released a dog to bite or scratch you
9. Forced you to have sex
10. Threatened to use a gun, knife, or other weapon against you
11. Used a knife to cut you
12. Shot a gun at you

Two additional questions were used to assess coercion, a hallmark of the trafficking experience: (1) ‘While you were in this situation, did anyone threaten to hurt you’? (yes/no) and (2) ‘During this time did anyone threaten to hurt your family or someone you care about’? (yes/no). These questions are also frequently used in the studies of interpersonal violence (Zimmerman *et al*., 2003; Zimmerman *et al*., 2006; Kelly and Johnson, 2008).

### Statistical analyses

All analyses accounted for the two-stage cluster sampling and the likely correlation of outcomes within a given sampled service using the variable post-trafficking service organisation as the cluster variable. We first examined frequency distributions and conducted bivariate analyses with cross-tabulations using Rao-Scott chi-square tests to assess associations between indicators of violence and coercion and indicators of anxiety, depression and PTSD status among females and males. We assessed the relationship of sex with each of the individual indicators of violence and coercion indicating different experiences of these acts in females and males, therefore we proceeded to conduct separate analyses by sex (Iglesias-Rios *et al*., 2018).

We used a person-centred statistical framework, employing mixture modelling to identify latent (unobserved) population subgroups from our observed measures of violence and coercion (Muthén and Muthén, 1998–2017; Muthén and Muthén, 2000; Lanza and Cooper, 2016). First, we used LCA modelling to identify latent classes of participants with distinct patterns of violence and coercion. The goal of the LCA was to identify the smallest number of latent classes that described meaningful patterns in the observed categorical indicators of violence and coercion. We examined 9 and 7 dichotomous indicators for females and males, respectively. We excluded indicator variables with small sample sizes (<30) to avoid convergence problems in the models. LCA parameters were estimated by maximum likelihood using an expectation-maximisation (EM) procedure. Missing data on the latent class indicators were handled by this procedure, with data assumed to be missing at random (Muthén and Muthén, 1998–2017).

To fit the LCA model, we started with a one class model and proceeded to test models including up to four classes. The model goodness of fit was assessed using the following information criteria: (1) Akaike Information Criterion (AIC), (2) Schwarz's Bayesian Information Criterion (BIC), (3) the sample-size-adjusted (BIC) and (4) the consistent Akaike Information Criterion (CAIC). Further, we assessed the entropy of each model, which is used as an indicator of the quality of the class separation. Entropy generally reflects the overall ability of the model to correctly classify subjects (Berlin *et al*., 2014).

Model fit was also examined using the average posterior probability of class membership for respondents assigned to their most likely class. Item response plots were employed to assess the patterns of conditional violence and coercion item-response probabilities and determine which items differentiated the latent classes. The Vuong-Lo-Mendell-Rubin adjusted likelihood ratio test was used to compare the model with *k* latent classes to the *k*-1 class solution; statistical significance (*p* < 0.05) suggests that the *k* class model provides a better fit than the model with *k*-1 classes (Muthén and Muthén, 1998–2017). Theory-driven information was considered in the interpretability and selection of the final latent class models for females and males. Models were examined for fit, parsimony, interpretability and separation of classes.

We used the multi-step mixture modelling approach of Bolck, Croon and Hagenaar, known as ‘BCH’ for categorical outcomes (Bolck *et al*., 2004; Asparouhov and Muthen, 2014), using separate models for females and males. The BCH statistical approach involves three main analytical steps. In the first step, the parameters of the LCA model are estimated without the distal outcomes. Second, the posterior probabilities of class membership based on this model are used to compute BCH weights for further analysis accounting for the uncertainty introduced by possible misclassification when estimating the model parameters providing a more robust model (Asparouhov and Bengt, 2014). Third, the calculated BCH weights were used to regress the binary outcomes (anxiety, PTSD and depression symptoms) on latent class membership and compare the classes in terms of the probability of each outcome. Clustering in the data related to the sampling strategy was managed by correcting the standard errors and chi-square tests of model fit using the TYPE  = COMPLEX analytical option and the CLUSTER option within the M*plus* software version 8.0 (Muthén and Muthén, 1998–2017). Robust standard errors were computed using the sandwich variance estimator.

## Results

### Sample characteristics of females and males

Table 1 presents the prevalence of violence and coercion experienced by survivors of trafficking by sex. A total of 569 (56.1%) females and 446 (43.9%) males participated in the study. Males were more likely to be exposed to various types of physical violence (e.g., slapped, dragged or beaten up). In contrast, females were more likely than males to suffer from sexual violence (35.0%). Coercion in the form of personal threats was more commonly reported by men and boys (46.2%) while receiving both personal and family threats was slightly more prevalent in females (14.6%).

**Table 1.** Prevalence of violence and coercion of formerly trafficked individuals by sex: the Study on Trafficking, Exploitation and Abuse in the Mekong Sub-region (STEAM), *n*  =  1015

Binary indicators of violence	Females	Males
	*n* (%)^a^	*n* (%)^a^
Total	569 (56.1)	446 (43.9)
Slapped you or threw something at you that could hurt you^b^		
Yes	125 (22.0)	163 (36.6)
Pushed or shoved you		
Yes	106 (18.6)	159 (35.7)
Hit you with a fist or with something else that could hurt you		
Yes	84 (14.8)	143 (32.1)
Kicked, dragged, or beat you up^b^		
Yes	87 (15.3)	112 (25.1)
Tied or chained you^b^		
Yes	20 (3.5)	6 (1.4)
Choked you on purpose^b^		
Yes	30 (5.3)	24 (5.4)
Burned you on purpose^b^		
Yes	3 (0.5)	6 (1.4)
Released a dog to bite or scratch you^c^		
Yes	3 (0.5)	8 (1.8)
Forced you to have sex^d^		
Yes	198 (35.0)	6 (1.4)
Threatened to use a gun, knife, or other weapon against you^c^		
Yes	53 (9.4)	103 (23.1)
Used a knife to cut you^e^		
Yes	14 (2.5)	25 (5.6)
Shot a gun at you^f^		
Yes	1 (0.2)	13 (2.9)
Receiving threats during trafficking		
None	345 (60.6)	192 (43.1)
Personal threats	141 (24.8)	206 (46.2)
Both, personal and family threats	83 (14.6)	48 (10.8)

a Column percentages.

b One missing for females.

c Two missing for females.

d Three missing for females and three missing for males.

e One missing for females.

f One missing for females.

### Latent class models for females and males

Table 2 presents the fit indices for the two-class models selected for females and males. Even though fit indices were smaller for the three-class models, high entropy in females (0.94) and males (0.91), consideration of the Vuong-Lo-Mendell Rubin Likelihood Ratio test, item response plots and theory suggested that the more parsimonious two class models were more appropriate. Online supplementary Figs 1 and 2 display item-response plots to describe the model-based probability of a positive response to each of the indicators of violence and coercion, enabling discernment of which items differentiated the latent classes. For females, salient features of violence were sexual violence, coercion and physical violence. In contrast, physical violence and personal coercion were common in males.

**Table 2.** Fit indices of a two latent class analysis models of violence and coercion among trafficking survivors female and males, *n*  =  1015

Index	Females (*n* = 569)			Males (*n* = 446)		
	II Classes	III Classes	IV Classes	II Classes	III Classes	IV Classes
Log-likelihood:	−1787.433	−1726.321	−1712.063	−1403.929	−1366.058	−1351.498
AIC^a^	3616.867	3516.641	3510.127	2841.858	2784.117	2772.997
BIC^b^	3708.088	3655.645	3696.914	2911.563	2890.725	2916.508
Adjusted BIC	3641.423	3554.060	3560.408	2857.612	2808.2120	2805.4330
Entropy	0.94	0.83	0.76	0.91	0.82	0.87
Vuong-Lo-Mendell Rubin likelihood ratio test (*p*-value)	0.07	0.42	0.72	0.12	0.40	0.55

a Akaike information criterion.

^b^Bayesian information criterion.

Table 3 displays conditional probabilities of violent acts and coercion within the given two latent classes with their standard errors, as well as latent class probabilities that show the prevalence of specific patterns of violence and coercion within the sampled population. For females, the two-class solution was informed by previous trafficking research and defined as ‘severe sexual and physical violence, and coercion (class I)’ and ‘sexual violence and coercion (class II)’ that characterised 20% and 80% of the sampled female population, respectively. Females in ‘class I’ experienced high probabilities (⩾60%) of exposure to a wide range of acts of physical violence, sexual violence (69%), threats with weapons (36%) and coercion (61% for personal threats and 31% for both personal and family threats). The pattern of ‘sexual violence and coercion (class II)’ was experienced by the vast majority of females (80%) with a relatively lower likelihood of sexual violence (27%) and coercion in the form of personal (16%) and both personal and family threats (10%).

**Table 3.** Two-class model of violence and coercion of formerly trafficked females and males, *n*  =  1015

Latent class prevalence based on the estimated model (%)	Females (*n* = 569)				Males (*n* = 446)			
		Class I Severe sexual and physical violence and coercion		Class II Sexual violence and coercion		Class I Severe physical violence and coercion		Class II Personal coercion
	0.20		0.80		0.41		0.59	
	Item response probability	s.e.^a^	Item response probability	s.e.	Item response probability	s.e.	Item response probability	s.e.
Violence and coercion indicators								
Slapped you or threw something at you that could hurt you	0.90	0.06	0.05	0.03	0.84	0.04	0.04	0.04
Pushed or shoved you	0.75	0.09	0.05	0.02	0.78	0.05	0.07	0.05
Hit you with a fist or with something else that could hurt you	0.67	0.10	0.02	0.01	0.75	0.03	0.02	0.01
Kicked, dragged, or beat you up	0.75	0.09	0.01	0.005	0.61	0.03	0.006	0.01
Tied or chained you	0.14	0.03	0.01	0.007	−	−	−	−
Choked you on purpose	0.25	0.04	0.005	0.006	0.13	0.01	0.005	0.01
Forced you to have sex	0.69	0.15	0.27	0.10	−	−	−	−
Threatened to use a gun, knife, or other weapon against you	0.36	0.05	0.03	0.01	0.50	0.04	0.05	0.05
Receiving threats during trafficking								
Personal threats	0.61	0.07	0.16	0.03	0.80	0.04	0.23	0.10
Both, personal and family threats	0.31	0.09	0.10	0.06	0.13	0.03	0.09	0.06

a Standard error.

The patterns of violence that characterised the trafficked male population were defined by ‘severe physical violence and coercion (class I, 41%)’ and ‘personal coercion (class II, 59%)’. For men and boys classified as exposed to ‘severe physical violence and coercion (class I)’, more than 60% had a high likelihood of reporting severe acts of physical violence, threats with weapons (50%) and personal threats (80%). In contrast, only 10% of men and boys were classified as being ‘personally coerced (class II)’.

### Test of prevalence equality for anxiety, PTSD and depression symptoms across latent classes in females and males using the BCH procedure

**Fig. 1.**
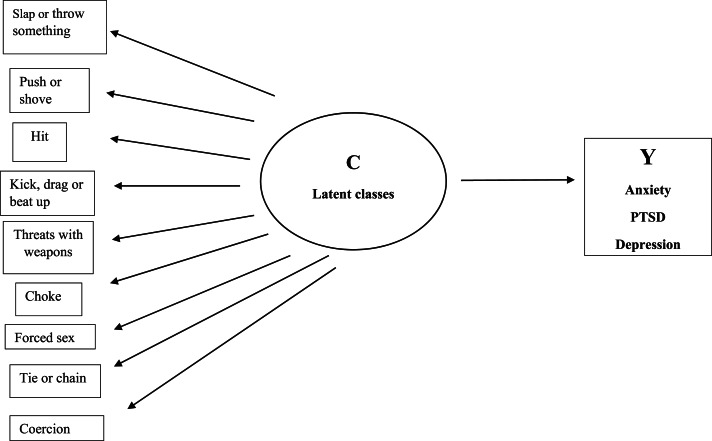
Finite mixture model diagram with binary latent class indicators of violence and coercion with binary outcomes: anxiety, post-traumatic stress disorder, and depressive symptoms. The arrow from C to Y (binary dependent variable) indicates that the intercept of Y varies across the classes of C. This corresponds to the logistic regression of Y on C classes.

Figure 1 illustrates the mixture model of the associations of the latent class variable with the binary outcomes (anxiety, PTSD, depression). We inspected missing data patterns and determined they were missing at random, given the relatively low number of missing data (e.g., one female and one male were missing for anxiety, depression and PTSD) we allowed for listwise deletion in the analysis.

Tests of prevalence equality for each of the distal outcomes and latent class membership for females and males are shown in Table 4. More than half of the sample of females in the ‘severe sexual and physical violence and coercion’ class had symptoms of anxiety (55%) and depression (79%). Similarly, 48% of women and girls in this class had PTSD symptoms. A majority of females classified as exposed to ‘sexual violence and coercion’ had symptoms of depression (61%) and about a third each had symptoms of anxiety (37%) and PTSD (31%). There were statistically significant differences in the prevalence of each of the three mental health outcomes between the two classes in females. Almost half of the males in the ‘severe physical violence and coercion’ class reported symptoms of anxiety (48%) and PTSD (46%), and more than half of males (59%) in this class had symptoms of depression. Among males classified as being exposed to ‘personal coercion’, approximately 45% had symptoms of anxiety, 39% had PTSD symptoms and 56% had symptoms of depression. However, there were no statistically significant differences between the classes and across each of the mental health outcomes in males.

**Table 4.** Tests of prevalence equality for the distal mental health outcomes (anxiety, depression and post-traumatic stress disorder symptoms) across latent classes in females and males using the BCH^a^ procedure, *n*  = 1015

	Females (*n* = 569)					
	Anxiety		PTSD^b^		Depression	
	%	s.e.^c^	%	s.e.	%	s.e.
Severe sexual and physical violence and coercion (class I)	55.0	6.0	48.0	0.10	79.0	0.08
Sexual violence and coercion (class II)	37.0	6.0	31.0	0.07	61.0	0.03
Overall chi-square (class I *v*. class II), *p*-value	*χ^**2**^*** = ** 26.33, *p* < 0.0001		*χ^**2**^*** = ** 4.03 *p* = 0.05		*χ^**2**^*** = ** 3.87 *p* = 0.05	
	Males (*n* = 446)					
	Anxiety		PTSD		Depression	
	%	s.e.	%	s.e.	%	s.e.
Severe physical violence and coercion (class I)	48.0	0.17	46.0	0.15	59.0	0.17
Personal coercion (class II)	45.0	0.11	39.0	0.12	56.0	0.10
Overall chi-square (class I *v*. class II), *p*-value	*χ^**2**^*** = ** 0.03 *p* = 0.86		*χ^**2**^*** = ** 0.12 *p* = 0.73		*χ ^**2**^*** = ** 0.02 *p* = 0.88	

a Bolck, Croon, and Hagenaar.

b Post-traumatic stress disorder symptoms.

c Standard error.

### Finite mixture gender-specific models

Table 5 displays estimates from the fitted mixture model for the gender-specific associations between latent class membership and each of the mental health symptoms. Females exposed to ‘severe violence (including physical and sexual violence) and coercion’ had a two-fold increase in the odds of anxiety (OR =  2.10; 95% CI: 1.57–2.81) and PTSD (OR =  2.07; 95% CI: 1.03–4.17) symptoms when compared with females exposed to ‘sexual violence and coercion (personal and family threats)’. Males exposed to ‘severe physical violence and coercion’ had higher odds of PTSD and depression symptoms relative to males exposed to ‘personal coercion’, but the associations were not statistically significant.

**Table 5.** Estimated mixture logistic regression analyses of the latent classes and anxiety, post-traumatic stress disorder and depressive symptoms using a BCH^a^ procedure, *n*  =  1015

	Anxiety		PTSD^b^		Depression	
	OR	95% CI	OR	95% CI	OR	95% CI
Females						
Sexual violence and coercion (reference)	1.0		1.0		1.0	
Severe sexual and physical violence and coercion	2.10	1.57–2.81	2.07	1.03–4.17	2.48	0.85–7.25
Males						
Personal coercion (reference)	1.0		1.0		1.0	
Severe physical violence and coercion	0.93	0.52–1.66	1.35	0.25–7.33	1.14	0.21–6.28

a Bolck, Croon and Hagenaar.

b Post-traumatic stress disorder symptoms.

## Discussion

This is the first study on human trafficking survivors to use a LCA with a multi-step mixture modelling (BCH) approach to further understand the complex gender patterns of violence and coercion and its association with the mental health of trafficking survivors exploited in various labour sectors. Consistent with the scarce evidence from gender-specific studies of human trafficking, we found that both females and males experienced severe forms of violence and coercion. The results of the LCA revealed a more complex picture of what constitutes violence and coercion for female and male trafficking survivors, in contrast to previous studies using single (e.g., ‘sexual violence only’) or binary (yes/no) measures of violence (Ottisova *et al*., 2016).

Our results showed a substantially elevated prevalence of symptoms of depression in females who suffered from ‘severe sexual and physical violence and coercion (class I, 79%)’ and those exposed to ‘sexual violence and coercion (class II, 61%)’. The prevalence of anxiety and PTSD symptoms was also considerably higher for class I in females. We also observed differences between the latent classes (class I *v*. II) in females with particularly elevated prevalence of anxiety, PTSD and depressive symptoms among those exposed to multiple acts of violence and coercion (class I). For males, more than half of the sample exposed to ‘severe physical violence and coercion’ (class I, 59%) had depression and almost 50% of men and boys in ‘class I’ had anxiety and PTSD symptoms. For females and males, this considerably higher prevalence of anxiety, PTSD and depression were similar to that reported among war-refugees and torture survivors (Lindert *et al*., 2009; Bogic *et al*., 2015; Ibrahim and Hassan, 2017).

We found a two-fold increase in the odds of anxiety and PTSD symptoms in females classified as being exposed to ‘severe sexual, and physical violence and coercion (threats with weapons, personal threats, and both personal and family threats)’ compared with females exposed to ‘sexual violence and coercion’. These findings are aligned with trauma research that reports that regardless of age, experiencing multiple or repeated exposures to traumatic events increases the risk of psychiatric morbidity, specifically anxiety, PTSD and depression (Suliman *et al*., 2009; Shalev *et al*., 2017). Our finding that sexual violence and coercion was highly prevalent in females is not unexpected, as they are deeply intertwined with gender inequality and human

trafficking is known to be highly gendered (World Health Organization, 2002; Anderson, 2009; International Labour Office, 2017).

For males, the associations for PTSD and depression symptoms were elevated among those who were exposed to ‘severe physical violence and coercion’, but did not differ across classes. It is plausible that we failed to detect differences between classes or that the pattern of violence for males is indeed defined solely as ‘severe physical violence and coercion’. Further research with larger sample sizes is needed as we had to exclude some categorical ‘violent’ indicators (e.g., sexual violence) due to small sample sizes. Yet results showed that besides physical violence, being threatened with weapons and experiencing personal threats were prevalent among men and boys classified in the ‘severe violence and coercion’ class. Our findings also indicated differences in the patterns of coercion for females and males. Females experienced ‘personal threats’, both ‘personal and family threats’, and ‘threats with weapons’ while for males, ‘personal threats only’ and ‘threats with weapons’ were more characteristic of their trafficking experience.

The results of the study have important implications for policy, research and mental health treatments. Our results highlight the need for considering how different patterns of violence and coercion affect the mental health of female and male trafficking survivors. These findings have important implications for survivors since exposure to multiple types of violence is associated with psychiatric disorders and physical morbidity, which could be particularly harmful for developing children and adolescents (World Health Organization, 2002; Finkelhor *et al*., 2011; Iglesias-Rios *et al*., 2018). Our findings could be valuable to understand differences in treatment needs, to employ targeted mental health interventions and assess which interventions would be more beneficial for specific groups of the trafficked population.

Our results highlight the importance of developing more comprehensive measures to capture the complexity and the effects of multiple acts of violence and coercion in this population. Measures could include frequency and severity of violent acts, associated injuries, perpetrators' controlling behaviours, survivor relationship with perpetrator (e.g., stranger, family member) and survivor's perception of what constitutes violence and coercion.

Our study has some limitations. The study population is a sample of survivors of trafficking using post-trafficking services in several key world regions which limits the generalisability of the study results. We cannot establish the temporal relationship between violence and coercion with mental health due to the cross-sectional study design. Mental health symptoms were assessed with validated screening instruments and not diagnostic tools. Therefore, the overestimation of the prevalence of mental health outcomes is possible. It is also plausible that trafficked individuals under the most severe forms of slavery are the ones with worse mental health and may be less likely to be reached in post-trafficking services. In this case, our calculations of the prevalence ratio will be underestimated.

Future research is needed to assess if the latent classes observed in this study apply to other country settings with different social gender roles and norms. Longitudinal studies are needed to evaluate the course of the effects of these patterns of violence and coercion in relation to mental health over time to inform the provision of services and mental health treatment for survivors. For instance, delayed or late onset of PTSD tends to appear at least 6 months after a post-trauma adjustment period in which diagnostic criteria was initially absent (American Psychiatric Association, 2013). In conclusion, this study demonstrates the importance of considering the patterns of violence and coercion and their relationships with mental health when implementing health services for female and male trafficking survivors.

## Supplementary material

For supplementary material accompanying this paper visit http://dx.doi.org/10.1017/S2045796019000295. click here to view supplementary material

## Availability of data and material

The datasets generated during and/or analysed during the current study are not publicly available due to safety concerns and the confidentiality agreement obligations with study participants. Data are available to qualifying researchers upon reasonable request from the Principal Investigator of STEAM (Dr Ligia Kiss).

## References

[ref1] American Psychiatric Association (2013) Diagnostic and Statistical Manual of Mental Disorders: DSM-5. Washington, DC: American Psychiatric Publishing.

[ref2] Anderson KL (2009) Gendering coercive control. Violence Against Women 15, 1444–1457. 19834070 10.1177/1077801209346837

[ref3] Asparouhov T and Bengt M (2014) Auxiliary variables in mixture modeling: Using the BCH method in Mplus to estimate a distal outcome model and arbitrary secondary model. p. 22. Mplus Web Notes: No. 21.

[ref4] Asparouhov T and Muthen B (2014) Auxiliary variables in mixture modeling: three-step approaches using mplus. Structural Equation Modeling-A Multidisciplinary Journal 21, 329–341.

[ref5] Berlin KS, Williams NA and Parra GR (2014) An Introduction to latent variable mixture modeling (part 1): overview and cross-sectional latent class and latent profile analyses. Journal of Pediatric Psychology 39, 174–187. 24277769 10.1093/jpepsy/jst084

[ref6] Bogic M, Njoku A and Priebe S (2015) Long-term mental health of war-refugees: a systematic literature review. BMC International Health and Human Rights 15, p.29. 10.1186/s12914-015-0064-9 PMC4624599 26510473

[ref7] Bolck A, Croon M and Hagenaars J (2004) Estimating latent structure models with categorical variables: one-step versus three-step estimators. Political Analysis 12, 3–27.

[ref8] Derogatis LR, Lipman RS, Rickels K, Uhlenhuth EH and Covi L (1974) The Hopkins Symptom Checklist (HSCL): a self-report symptom inventory. Behavioral Science 19, 1–15. 4808738 10.1002/bs.3830190102

[ref9] Finkelhor D, Turner H, Hamby S and Ormrod R (2011). Polyvictimization: children's exposure to multiple types of violence, crime, and abuse. In Office of Justice Programs (ed.), Juvenile Justice Bulletin (NCJ 235504). Washington, DC: U.S. Department of Justice, pp. 1–11.

[ref10] Garcia-Moreno C, Jansen HA, Ellsberg M, Heise L and Watts CH (2006) Prevalence of intimate partner violence: findings from the WHO multi-country study on women's health and domestic violence. The Lancet 368, 1260–1269. 10.1016/S0140-6736(06)69523-8 17027732

[ref11] Hopper E and Hidalgo J (2006) Invisible chains: psychological coercion of human trafficking victims. Intercultural Human Rights Law Review 1, 185–210.

[ref12] Hossain M, Zimmerman C, Abas M, Light M and Watts C (2010) The relationship of trauma to mental disorder among trafficked and sexually exploited girls and women. American Journal of Public Health 100, 2442–2449. 20966379 10.2105/AJPH.2009.173229 PMC2978168

[ref13] Ibrahim H and Hassan CQ (2017) Post-traumatic stress disorder symptoms resulting from torture and other traumatic events among Syrian Kurdish refugees in Kurdistan Region, Iraq. Frontiers in Psychology 8, 1–8. 28265252 10.3389/fpsyg.2017.00241 PMC5316552

[ref14] Iglesias-Rios L, Harlow SD, Burgard SA, Kiss L and Zimmerman C (2018) Mental health, violence and psychological coercion among female and male trafficking survivors in the greater Mekong sub-region: a cross-sectional study. BMC Psychology 6, 1–15. 30541612 10.1186/s40359-018-0269-5 PMC6292017

[ref15] International Labour Office (2017) Global Estimates of Modern Slavery: Forced Labour and Forced Marriage. Geneva, 64. https://www.ilo.org/global/publications/books/WCMS_575479/lang--en/index.htm (Accessed 12 September 2018).

[ref16] Kelly JB and Johnson MP (2008) Differentiation among types of intimate partner violence: research update and implication for interventions. Family Court Review 46, 476–499.

[ref17] Kiss L, Yun K, Pocock N and Zimmerman C (2015) Exploitation, violence, and suicide risk among child and adolescent survivors of human trafficking in the greater Mekong subregion. JAMA Pediatrics 169, e152278, pp. 1–8. 10.1001/jamapediatrics.2015.2278 26348864

[ref18] Kleijn WC, Hovens JE and Rodenburg JJ (2001) Posttraumatic stress symptoms in refugees: assessments with the Harvard Trauma Questionnaire and the Hopkins symptom checklist-25 in different languages. Psychological Reports 88, 527–532. 11351903 10.2466/pr0.2001.88.2.527

[ref19] Lanza ST and Cooper BR (2016) Latent class analysis for developmental research. Child Development Perspectives 10, 59–64. 31844424 10.1111/cdep.12163 PMC6914261

[ref20] Lindert J, von Ehrenstein OS, Priebe S, Mielck A and Brähler E (2009) Depression and anxiety in labor migrants and refugees–a systematic review and meta-analysis. Social Science & Medicine 69, 246–257. 19539414 10.1016/j.socscimed.2009.04.032

[ref21] Mollica RF, Wyshak G, de Marneffe D, Khuon F and Lavelle J (1987) Indochinese versions of the Hopkins Symptom Checklist-25: a screening instrument for the psychiatric care of refugees. The American Journal of Psychiatry 144, 497–500. 3565621 10.1176/ajp.144.4.497

[ref22] Mollica RF, Caspi-Yavin Y, Bollini P, Truong T, Tor S and Lavelle J (1992) The Harvard Trauma Questionnaire: validating a cross-cultural instrument for measuring torture, trauma, and posttraumatic stress disorder in Indochinese refugees. Journal of Nervous and Mental Disease 180, 111–116. 1737972

[ref23] Mollica RF, Donelan K, Tor S, Lavelle J, Elias C, Frankel M and Blendon RJ (1993) The effect of trauma and confinement on functional health and mental health status of Cambodians living in Thailand-Cambodia border camps. JAMA 270, 581–586. 8331755

[ref24] Mollica RF, Caspi-Yavin Y, Lavelle J, Tor S, Yang T, Chan S, Pham T, Ryan A and De Marneffe D (1996) Harvard trauma questionnaire (HTQ) manual: Cambodian, Lao, and Vietnamese versions. Torture 6(Suppl. 1), 19–33.

[ref25] Muthén LK and Muthén BO (1998–2017) Mplus User's Guide. Los Angeles, CA: Muthén & Muthén.

[ref26] Muthén BO and Muthén LK (2000) Integrating person-centered and variable-centered analyses: growth mixture modeling with latent trajectory classes. Alcoholism: Clinical and Experimental Research 24, 882–891. 10888079

[ref27] O'Donnell ML, Schaefer I, Varker T, Kartal D, Forbes D, Bryant RAA, Silove D, Creamer M, McFarlane A, Malhi G, Felmingham K, Van Hoof M, Hadzi-Pavlovic D, Nickerson A and Steel Z (2017) A systematic review of person-centered approaches to investigating patterns of trauma exposure. Clinical Psychology Review 57, 208–225. 28919323 10.1016/j.cpr.2017.08.009

[ref28] Ottisova L, Hemmings S, Howard LM, Zimmerman C and Oram S (2016) Prevalence and risk of violence and the mental, physical and sexual health problems associated with human trafficking: an updated systematic review. Epidemiology and Psychiatric Sciences 25, 317–341. 27066701 10.1017/S2045796016000135 PMC7137602

[ref29] Shalev A, Liberzon I and Marmar C (2017) Post-traumatic stress disorder. The New England Journal of Medicine 376, 2459–2469. 28636846 10.1056/NEJMra1612499

[ref30] Suliman S, Mkabile SG, Fincham DS, Ahmed R, Stein DJ and Seedat S (2009) Cumulative effect of multiple trauma on symptoms of posttraumatic stress disorder, anxiety, and depression in adolescents. Comprehensive Psychiatry 50, 121–127. 19216888 10.1016/j.comppsych.2008.06.006

[ref31] Turner-Moss E, Zimmerman C, Howard LM and Oram S (2014) Labour exploitation and health: a case series of men and women seeking post-trafficking services. Journal of Immigrant and Minority Health 16, 473–480. 23649665 10.1007/s10903-013-9832-6

[ref32] World Health Organization (2002) World Report on Violence and Health. (https://www.who.int/violence_injury_prevention/violence/world_report/en/) (Accessed 12 September 2018).

[ref33] World Health Organization & Pan American Health Organization (2012). Understanding and Addressing Violence Against Women: Human Trafficking. http://www.who.int/iris/handle/10665/77394 (Accessed 8 September 2018).

[ref34] Zimmerman C and Kiss L (2017) Human trafficking and exploitation: a global health concern. PLOS Medicine 14, e1002437, pp. 1–8. 29166396 10.1371/journal.pmed.1002437 PMC5699819

[ref35] Zimmerman C, Yun K, Shvab I, Watts C, Trappolin L, Treppete M, Bimbi F, Adams B, Jiraporn S, Beci L, Albrecht M, Bindel J and Regan L (2003)The Health Risks and Consequences of Trafficking in Women and Adolescents: Findings From a European Study. Including: Human Rights Analysis of Health and Trafficking and Principles for Promoting the Health Rights of Trafficked Women. London: London School of Hygiene & Tropical Medicine.

[ref36] Zimmerman C, Hossain M, Yun K, Roche B, Morison L and Watts C (2006) Stolen Smiles: A Summary Report on the Physical and Psychological Health Consequences of Women and Adolescents Trafficked in Europe. London School of Hygiene and Tropical Medicine. https://www.icmec.org/wp-content/uploads/2015/10/Stolen-Smiles-Physical-and-Psych-Consequences-of-Traffic-Victims-in-Europe-Zimmerman.pdf) (Accessed 12 June 2018).

[ref37] Zimmerman C, Hossain M, Yun K, Gajdadziev V, Guzun N, Tchomarova M, Ciarrocchi RA, Johansson A, Kefurtova A, Scodanibbio S, Motus MN, Roche B, Morison L and Watts C (2008) The health of trafficked women: a survey of women entering posttrafficking services in Europe. American Journal of Public Health 98, 55–59. 18048781 10.2105/AJPH.2006.108357 PMC2156078

