# Supplementary figures and images for: Patterns of violence and coercion with mental health among female and male trafficking survivors: a latent class analysis with mixture models
Source: Epidemiol Psychiatr Sci. 2019 May 30;29:e38. doi: 10.1017/S2045796019000295 (PMC7083520; doi:10.1017/S2045796019000295)

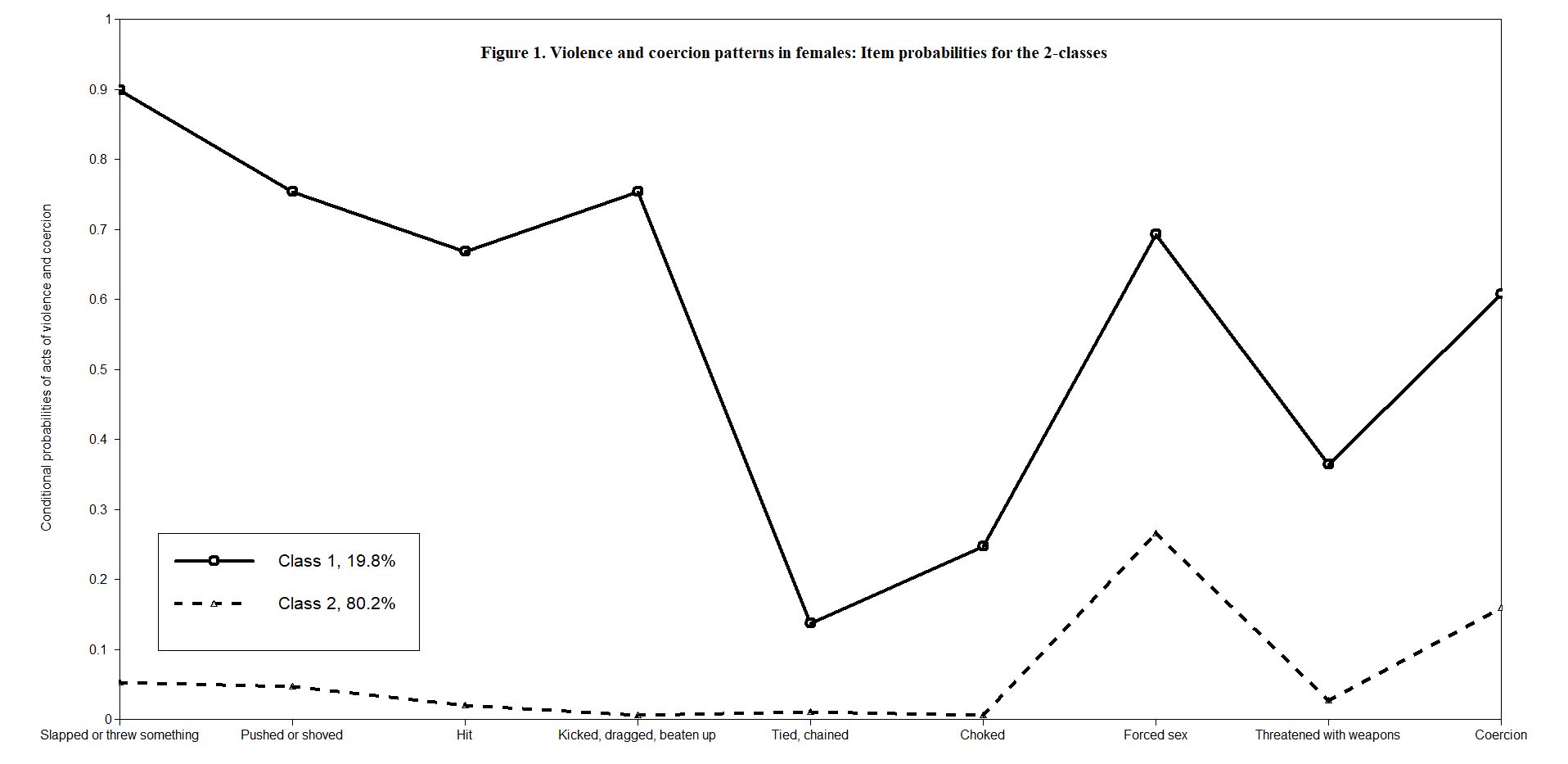

Supplement: Supplementary file 1 [file epssup.zip › S2045796019000295sup001.tiff]

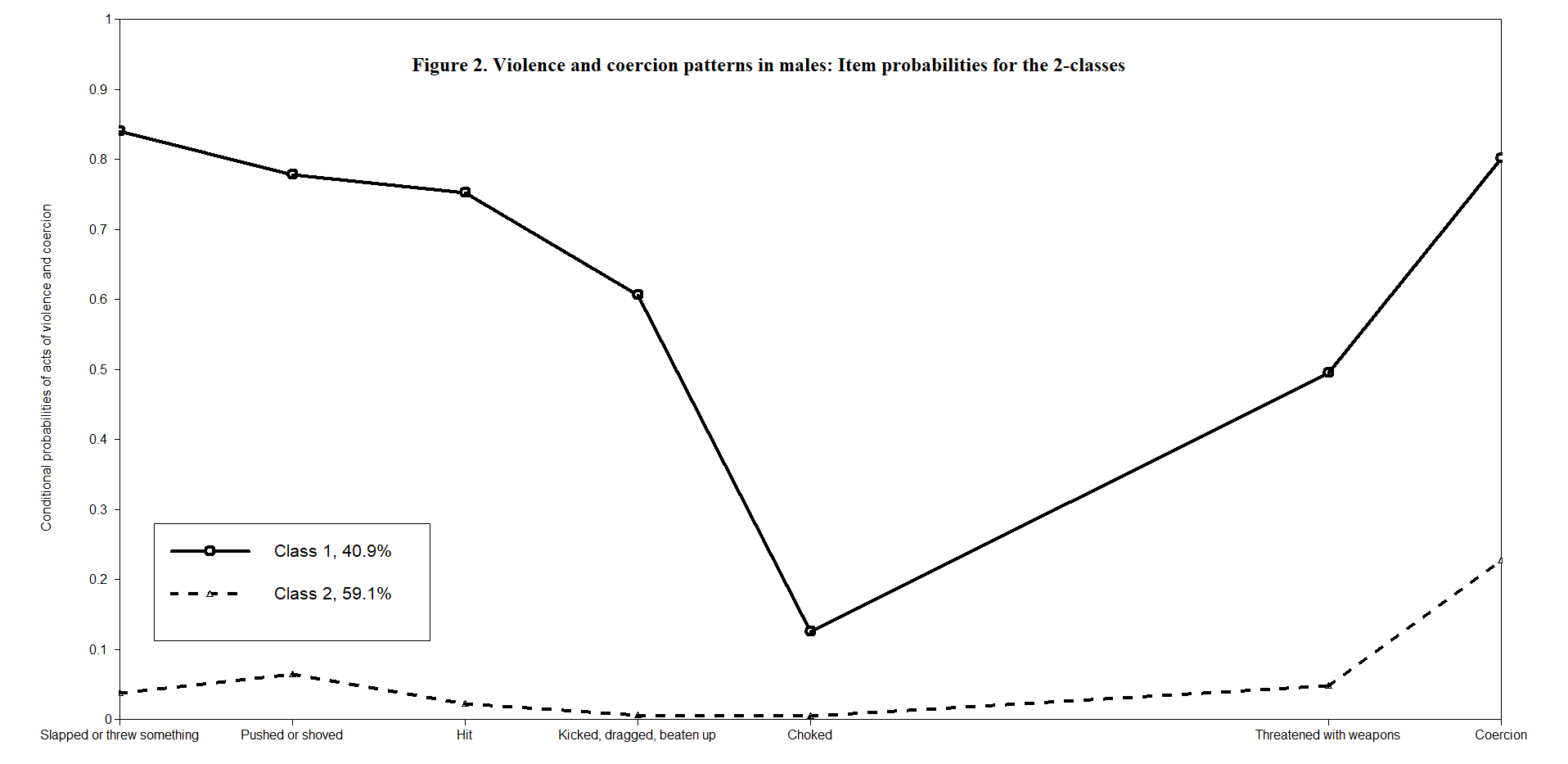

Supplement: Supplementary file 1 [file epssup.zip › S2045796019000295sup002.tiff]
